# Application of single cell multiomics points to changes in chromatin accessibility near calcitonin receptor like receptor and a possible role for adrenomedullin in the post-shock lung

**DOI:** 10.3389/fmed.2023.1003121

**Published:** 2023-04-11

**Authors:** Brandon E. Armstead, Chung Sunny Lee, Yaping Chen, Runping Zhao, Chun-Shiang Chung, Alger M. Fredericks, Sean F. Monaghan, Alfred Ayala

**Affiliations:** ^1^Lifespan-Rhode Island Hospital, Providence, RI, United States; ^2^Division of Surgical Research, Department of Surgery, Brown University, Providence, RI, United States; ^3^Pathobiology Graduate Program, Brown University, Providence, RI, United States; ^4^The Miriam Hospital, Providence, RI, United States; ^5^The Warren Alpert Medical School, Brown University, Providence, RI, United States

**Keywords:** shock, sepsis, trauma, single cell, multiomics, ALI, ARDS

## Abstract

**Introduction:**

Acute lung injury (ALI)/acute respiratory distress syndrome (ARDS) is a commonly occurring sequelae of traumatic injury resulting from indirect insults like hypovolemic shock and/or extrapulmonary sepsis. The high lethality rate associated with these pathologies outlines the importance of clarifying the “priming” effects seen in the post-shock lung microenvironment, which are understood to bring about a dysregulated or overt immune response when triggered by a secondary systemic infectious/septic challenge culminating in ALI. In this pilot project, we test the hypothesis that application of a single cell multiomics approach can elucidate novel phenotype specific pathways potentially contributing to shock-induced ALI/ARDS.

**Methods:**

Hypovolemic shock was induced in C57BL/6 (wild-type), PD-1, PD-L1, or VISTA gene deficient male mice, 8–12 weeks old. Wild-type sham surgeries function as negative controls. A total of 24-h post-shock rodents were sacrificed, their lungs harvested and sectioned, with pools prepared from 2 mice per background, and flash frozen on liquid nitrogen. *N* = 2 biological replicates (representing 4 mice total) were achieved for all treatment groups across genetic backgrounds. Samples were received by the Boas Center for Genomics and Human Genetics, where single cell multiomics libraries were prepared for RNA/ATAC sequencing. The analysis pipeline Cell Ranger ARC was implemented to attain feature linkage assessments across genes of interest.

**Results:**

Sham (pre-shock) results suggest high chromatin accessibility around calcitonin receptor like receptor (CALCRL) across cellular phenotypes with 17 and 18 feature links, exhibiting positive correlation with gene expression between biological replicates. Similarity between both sample chromatin profiles/linkage arcs is evident. Post-shock wild-type accessibility is starkly reduced across replicates where the number of feature links drops to 1 and 3, again presenting similar replicate profiles. Samples from shocked gene deficient backgrounds displayed high accessibility and similar profiles to the pre-shock lung microenvironment.

**Conclusion:**

High pre-shock availability of DNA segments and their positive correlation with CALCRL gene expression suggests an apparent regulatory capacity on transcription. Post-shock gene deficient chromatin profiles presented similar results to that of pre-shock wild-type samples, suggesting an influence on CALCRL accessibility. Key changes illustrated in the pre-ALI context of shock may allow for additional resolution of “priming” and “cellular pre-activation/pre-disposition” processes within the lung microenvironment.

## 1. Introduction

### 1.1. Severe shock as a driver of ALI/ARDS

The altered cellular microenvironment resultant from severe blood loss has proven to be an important pre-dispositional contributor to downstream injury or organ failure (1–3). This is often characteristic of individuals who have undergone traumatic injury and reduced systemic blood volume resulting in sustained reduced mean arterial blood pressure (hypovolemic/hemorrhagic shock). Shock initially primes a variety of organ compartments, especially the lung, and results in a hyper-sensitive state for all cells within these tissues, a.k.a., priming, predisposition, re-programming, innate immune memory, etc. (1, 4–6). Oftentimes, surgical intervention corrects the injuries associated with the traumatic event. However, damage to the bowel-a reservoir of pathogens capable of causing severe infections-may be disseminated during surgery. A septic event may occur, which is characterized as the host dysregulated immune response to systemic infection (7). This infectious challenge then triggers the priming seen after shock and may result in varying severe pathologies which may contribute to acute lung injury (ALI).

One form of ALI, acute respiratory distress syndrome (ARDS), is commonly seen in patients who have undergone shock and/or sepsis (1, 2). The high mortality associated with ARDS (approximately 40% lethality) further highlights the importance of additional characterization and phenotypic identification of the pre-disease state elicited by shock in the lung (8). While targeted therapeutics have yet to offer considerable benefits in treating ARDS patients, the post-shock lung microenvironment has provided an important landscape worthy of survey and putative therapeutic targeting.

### 1.2. ALI pre-disease states

Acute lung injury is resultant from a variety of pre-injury or pre-disease states all of which culminate in tissue damage and eventual organ failure (1, 2, 8). These contributing factors are often characterized as either direct or indirect sources of ALI (8). Pulmonary contusion, or injury to the lung itself is an example of direct ALI. Other sources include, aspiration on gastric/bowel contents and pneumonia. Indirect sources of ALI include extrapulmonary septic events, shock, and massive blood transfusion. Additional characterization of these causative factors to ALI provides further clarity on molecular mechanisms important to disease predisposition, and may further improve currently exasperated and somewhat ineffective treatment modalities (9, 10).

### 1.3. Novel methods in transcriptional and chromatin profiling

Single cell or single nuclei methods for analyzing the transcriptional milieu and chromatin landscape have provided useful tools for clarifying and validating phenotype specific alterations in gene expression (11–13). Transcriptional assessments through single cell methods alone offer useful and informative conclusions not available through the implementation of bulk sequencing methods (11, 14). However, there remains some ambiguity in these analyses. The confidence in accurate evaluation of relevant biological phenomena has necessitated use of other assays such as flow cytometry analysis or qPCR as validation steps. Another helpful approach has been use of the assay for transposase accessible chromatin (ATAC) sequencing (15). This allows for the identification of changes in chromatin availability to transcription factors and key regulatory proteins, which importantly bind promoter regions or enhancer/silencer elements of transcribed genes (16, 17). Open or accessible regions of chromatin are understood to be positions of these important regulatory interactions. Such regions influence transcription of genes upstream or downstream of their location. Therefore, implementation of ATAC sequencing allows for the identification of regulatory elements influencing expression of genes enriched and detected through RNA sequencing.

Generating both RNA and ATAC sequencing readouts from a single cell offers an extremely useful means of corroborating data. This was recently adopted as a multiomics technique providing information on correlation strength between accessible chromatin as well as transcript abundance at single cell resolution (18). Despite these advancements in technology, there remains the burden of further confirmation of multiomics datasets. However, through the novel incorporation of both transcriptomics and identification of chromatin landscape alterations, an initial level of confidence may be assigned to such analyses due to their tightly linked regulatory nature.

The most important aspect of the Cell Ranger ARC analysis pipeline used for this multiomics approach is the delineation of correlation strength between transcript abundance and accessible chromatin. These are referred to as linked features (19). Default pipeline settings allowed for the identification of links within 1 Mb. The stronger this relationship, the more likely an accessible locus possesses some regulatory capacity. Positive correlation beyond a pre-determined significance threshold aids in identifying enhancer elements, which drive transcription of linked genes. Anticorrelation is also detectable and occurs in the event of low transcript abundance and highly accessible chromatin, which indicates silencer elements that suppress transcription of linked genes. Silencers and enhancers are important regulatory genomic elements that will be discussed later. Both can be identified through this single cell multiomics approach.

### 1.4. Checkpoint proteins as immunomodulators of ALI

Signaling interactions between immunomodulatory receptors and ligands expressed across cellular phenotypes have recently been leveraged for immunotherapeutic benefits (20, 21). These regulators referred to as checkpoint proteins, have offered considerable benefits pertaining to tumor resolution and attenuating dysregulated immune responses leading to severe pathologies. Several of these proteins of particular importance serve as suppressors of the immune response. Following the initial signals required for lymphocyte activation which include antigen presentation *via* MHC I/II to the T-cell receptor, and co-stimulation by B-7 family/CD28 interactions, checkpoint proteins serves to either exacerbate or suppress subsequent immune activation.

Interactions between programmed cell death receptor 1 (PD-1) and its ligands (PD-L1/L2) importantly serve to suppress the immune response by blocking the activity of several key adaptor molecules, which normally act to stimulate T-cell activation (22–25). PD-1 is mainly expressed on lymphocytes and antigen presenting cells, but also across the endothelium/epithelium. These checkpoint proteins have been targeted clinically for oncogenic clearance (26), and an understanding of these benefits may be useful for other maladies of immune dysregulation pertaining to ALI/ARDS.

Another checkpoint protein providing putative utility in curtailing an aberrant immune response is the B-7 Ig superfamily member V-domain immunoglobulin suppressor of T-cell activation (VISTA) (27), which is primarily expressed on hematopoietic lineages (20, 28, 29). Myeloid populations possess higher VISTA expression than lymphoid groups, but among lymphocytes it is most highly expressed on regulatory T-cells. Interestingly, VISTA behaves as either a receptor or ligand and the outcome of its signaling is strongly tied to the cell and tissue specific context of these interactions. Ultimately, VISTA signaling suppresses the immune response and may drive other outcomes related to the development of ALI. Important therapeutic potential may be available through targeted stimulation or antagonism of VISTA signaling in the pre-development of lung inflammation, endothelial barrier dysfunction and/or fibrosis.

### 1.5. Disparate sepsis survival outcomes in the context of specific gene deficiencies

Sepsis is the most commonly seen pre-disease state for ALI (8). It is important to further characterize this relevant disease phenotype, especially in the context of checkpoint proteins and their impact on sepsis mortality. In experimental sepsis induced *via* the cecal ligation and puncture method, C57BL/6 mice deficient in PD-1 or PD-L1 expression relative to wild-type counterparts display improved survival (30, 31). However, mice deficient in VISTA expression are imparted a survival disadvantage relative to the wild-type background (32). Despite the shared co-inhibitory nature of their signaling, these distinct mortality events in the context of experimental sepsis offer novel insights on the complex nature of checkpoint protein biology and specific impacts on pre-ALI disease states worthy of additional clarification. Shock presents another pre-dispositional insult for the development of ALI. Such checkpoint protein deficiencies are important and may create unique microenvironments within the lung concurrent with altered priming states.

### 1.6. Hypothesis

**FIGURE 1:**
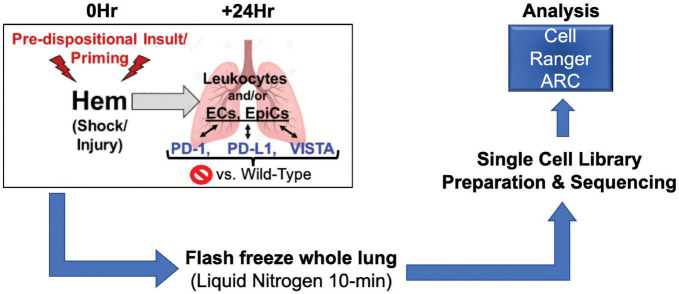
Illustrates workflow involved in data acquisition. Surgical techniques, sample harvesting, sequencing library preparation, and data analysis make up this process (*N* = 2/group).

Taken together, we posit that the application of a single cell multiomics approach should allow us to begin to elucidate novel phenotype specific pathways potentially contributing to shock-induced priming/reprograming/predisposition to the development of ALI/ARDS in the face of a subsequent septic insult. Presented here is a multiomics analysis of wild-type vs. gene deficient murine lungs in the context of hemorrhage (hem) computed through Cell Ranger ARC (33), as depicted in Figure 1.

## 2. Results

### 2.1. Unsupervised analysis

**FIGURE 2:**
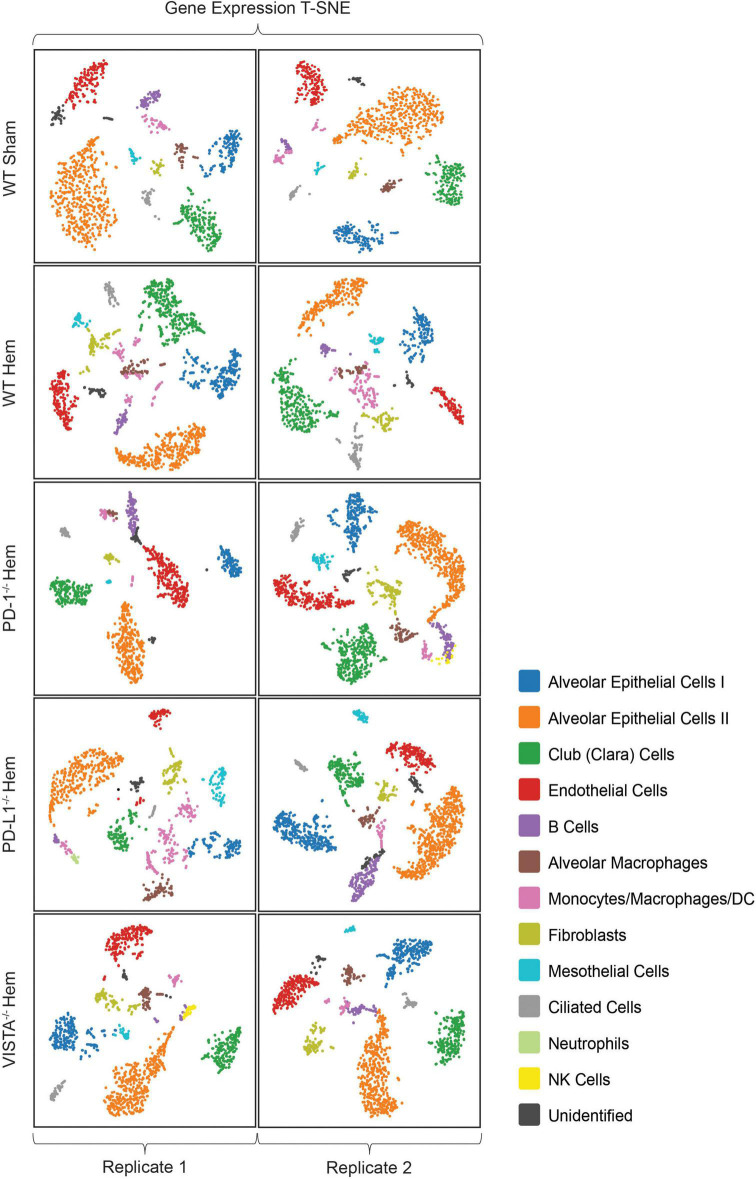
All samples in this multiomics approach are presented here in T-SNE plot format from Loupe Browser data visualization software. Wild-type sham/hemorrhaged as well as gene deficient hemorrhaged profiles are depicted. Samples are bracketed by individual biological replicates. Colored datapoints are clustered according to similarities in transcriptional profiles (*N* = 2/group).

Following implementation of the analysis pipeline Cell Ranger ARC on all 10 multiomics datasets, graph based clustering results were filtered/re-clustered based on cells falling within the linear distribution cut-off range of unique molecular identifiers (UMI’s), features per barcode and a threshold of mitochondrial reads through the Loupe Browser data visualization platform (34). Clusters from the gene expression plot were identified based on similar patterns from referenced murine lung NGS (35–37) genes and others found in the online reference BioGPS (38) (Supplementary Table 1). Sample plots are shown in T-SNE (39) format, as presented in Figure 2. Identities included type 1 (ATI) and type II (ATII) alveolar epithelial cells, club (clara) cells, endothelial cells, B cells, alveolar macrophages, a mixed population of monocytes/macrophages/dendritic cells, fibroblasts, mesothelial cells, ciliated cells and an unidentified population of cells with overlapping expression from both endothelial and ATII cells. NK cells were only detected as a sizable cluster amongst PD-1^–/–^ hem (2) and VISTA^–/–^ hem (1). Neutrophils were unexpectedly detected in PD-L1^–/–^ hem (1), as they are polymorphonuclear cells that should not have been captured. The sample preparation workflow applied here targets single nuclei. Neutrophils have proven difficult to detect in other single cell methods due to several factors, including their relatively low content of RNA and high amount of RNases (40).

**FIGURE 3:**
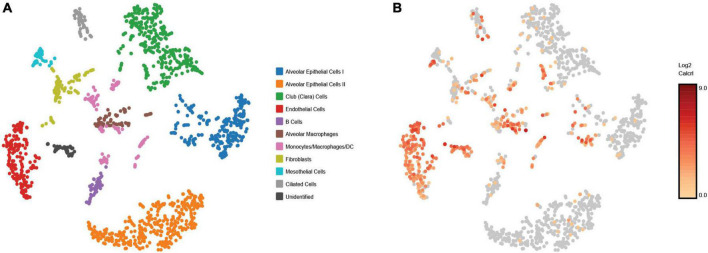
Depicts graph based projections **(A)** for all clusters in a representative sample [wild-type hem (1)], and its log2 fold change expression profile **(B)** for *Calcrl.*

**FIGURE 4:**
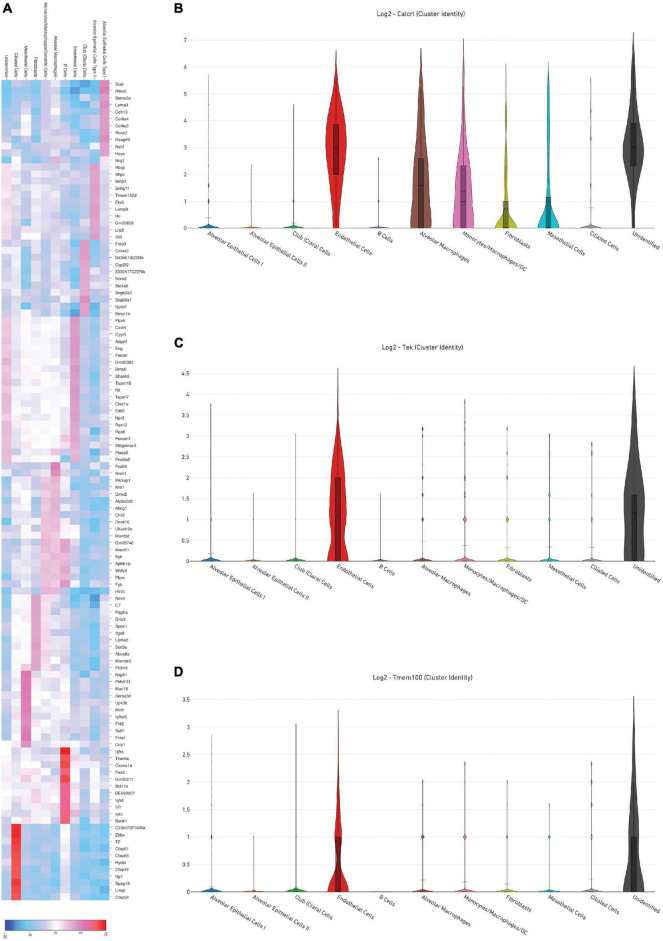
**(A)** Depicts a heat map of top significant genes upregulated per cluster (log2 fold change) in a representative sample [wild-type hem (1)]. Violin plots show relative log2 fold change expression profiles for markers used to identify endothelial cells in this sample: *Calcrl* **(B)**, *Tek* **(C)**, and *Tmem100* **(D)**.

Representative images from sample WT hem (1) are shown to depict unique cellular identities (Figure 3A) alongside the log2 fold change expression profiles of Calcitonin receptor like receptor *(Calcrl)* (Figure 3B), one of several markers used to identify endothelial cells. A heat map from this sample (Figure 4A) depicts some of the top upregulated genes expressed per cluster identity (log2 fold change) shown in T-SNE format, as well as *Calcrl* (Figure 4B), and the remaining endothelial markers *Tek* (Figure 4C) and *TMEM100* (Figure 4D) in violin plots.

### 2.2. Feature linkage assessment

In an attempt to discover genes with consistent expression and chromatin accessibility between biological replicates in the sham (wild-type) background, profiles for targets of interest were compared. These were then compared to hemorrhaged wild-type samples with distinct alterations in accessibility, and similarly for consistency between replicates. The third approach was surveying accessibility and expression across hemorrhaged samples gene deficient in global PD-1, PD-L1, or VISTA expression. Replicate consistency was again taken into account.

**FIGURE 5:**
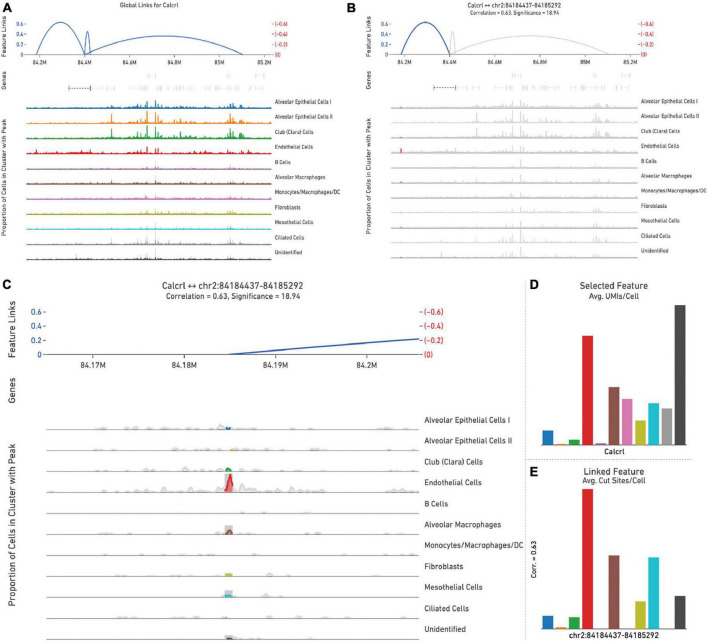
All feature links are depicted (within 1 Mb of *Calcrl*) **(A)** for representative sample wild-type hem (1). A single feature link is highlighted **(B)**, then shown at higher resolution at this specific locus chr2:84184437-84185292 **(C)**. The genomic location of *Calcrl* is denoted by the bracketed dashed line below the linkage arcs. **(D)** Displays average number of *Calcrl* unique molecular identifiers (UMIs), or cut sites per cell at this locus **(E)** for cells within each identified phenotype, presented in bar graph format.

Representative images from one sample (Figure 5) are shown to illustrate global links for *Calcrl* (Figure 5A), a single (highlighted) link (Figure 5B), and higher resolution at this specific chromosome locus of accessibility (Figure 5C). The average number of single cell RNA-seq UMI’s detected per cluster for *Calcrl* (Figure 5D), and average number of single cell ATAC-seq cut sites per cell at this specific locus (Figure 5E) are presented in bar graph format for all cells identified in this sample. The relative strength of correlation between linked features is indicated by the height of blue linkage arcs (19). Higher amplification arcs indicate strong correlation between transcript abundance and detectable accessible chromatin. Below each set of linkage arcs is pertinent gene information. Below this, is a representation of peak accessibility colored by the associated cluster. The height of each peak indicates the proportion of cells with accessible chromatin at that particular locus.

**FIGURE 6:**
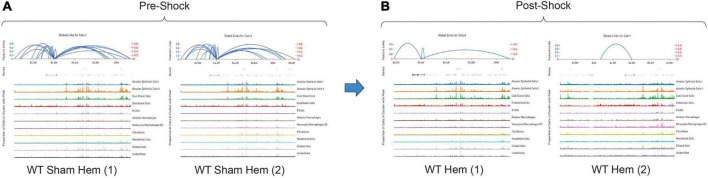
Feature linkage assessment of wild-type pre **(A)** and post-shock **(B)** lung microenvironments of *Calcrl.* Blue linkage arcs are displayed at the top of each sample plot; relative strength of correlation between accessible loci and *Calcrl* enrichment is conveyed *via* arc height. Colored peak information below arcs displays accessibility based on cells in individual clusters. The abundance of cells with accessible chromatin is represented by peak heights (*N* = 2/group).

**FIGURE 7:**
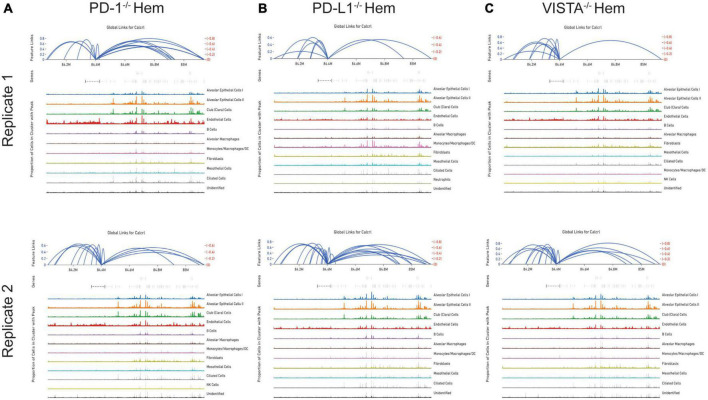
Feature linkage assessment of hemorrhaged gene deficient backgrounds. PD-1^–/–^ **(A)**, PD-L1^–/–^ **(B)** and VISTA^–/–^ **(C)**. Data is displayed as represented in Figure 6.

By totaling the number of feature links for specific genes in a given sample, the process of clarifying regulatory networks is somewhat expedited. *Calcrl* was found to be significantly enriched across clusters in both sham samples. Importantly, there was a similar number of feature links between replicates, with 18 in sample 1 (Figure 6A and Table 1) and 17 in sample 2. These loci were similarly positioned both upstream and downstream the *Calcrl* gene on Chromosome 2, and also exhibited positive correlation. In the hemorrhaged wild-type background, feature linkages were starkly reduced and consistent between replicates (Figure 6B and Table 1) as illustrated by 3 links and 1 link in samples 1 and 2, respectively. All were positively correlated. These data are also depicted in Supplementary Tables 2–5. In the context of specific gene deficiencies following hemorrhage shown in Figure 7 and Table 1, PD-1^–/–^ (Figure 7A) featured strong consistency between replicates with 15 and 13 feature links. PD-L1^–/–^ (Figure 7B) was less consistent, as shown by 8 and 22 feature links. VISTA^–/–^ featured somewhat greater consistency (Figure 7C) with 10 and 16 feature links. These were also all positively correlated; data are tabulated in Supplementary Tables 6–11.

**TABLE 1:** Total numbers of feature linkages are listed for each sample/replicate (*N* = 2/group).

	Total feature links				
**Sample**	**WT sham hem**	**WT hem**	**PD-1^–/–^ hem**	**PD-L1^–/–^ hem**	**VISTA^–/–^ hem**
Replicate 1	18	3	15	8	10
Replicate 2	17	1	13	22	16

While the hemorrhaged wild-type backgrounds displayed reduced feature links, individual deficiencies in costimulatory checkpoint proteins appeared to maintain high accessibility around this particular gene. These profiles appeared more similar to sham controls than hemorrhaged wild-type samples.

## 3. Discussion

### 3.1. Genomic regulatory elements and transcription

Distally dispersed genomic loci can influence the transcription of linked genes following the binding of relevant adaptor molecules (15). A conformational change occurs within the chromatin structure once these proteins bind to such regulatory regions (16, 17). Proteins bound to this locus may then interact with transcription factors bound to the promoter region of a relevant gene, further influencing its transcription. In this way, such structural changes allow the bound regulatory region to subsequently influence gene regulation. As mentioned in the description of feature linkage assessments, elements that increase or upregulate transcription are referred to as enhancers. Those that suppress transcription are known as silencers. The nature of key modifications at the chromatin level allows for increased availability of these regulatory loci to adaptor binding. Through silencer or enhancer activity and subsequent chromatin folding, linked genes undergo altered transcription. These important dynamics serve to further illustrate the relationship between chromatin accessibility and transcriptional networks.

### 3.2. Cellular phenotypes amongst lung datasets

A broad distribution of identities from referenced datasets were detected throughout the samples represented here (35–37). The frequencies at which these were found across each sample somewhat varied, although this analysis was completed by one individual. That may account for some of these apparent disparities between calls made in the determination of cluster identities. Additional heat maps are provided illustrating each sample and the top enriched genes per cluster (Supplementary Figures 1–10).

An inherent inability to identify a prevalent neutrophil (polymorphonuclear) signal amongst this dataset due to single nuclei workflow limitations, particularly in hemorrhaged samples where a high proportion of neutrophils is expected (1) has confounded certain aspects of this analysis, including an identification of their specific genomic alterations. This is with the exception of one sample detailed previously. The library preparation protocol for the datasets presented here relies on whole nuclei as an input material. Polymorphonuclear cells do not feature the typical single nucleus seen in other phenotypes (41) and, thus, are not expected to be captured through these methods due to sample sorting prior to library preparation.

### 3.3. *Calcrl* accessibility

As detected by the multiomics approach employed in this analysis, the receptor CALCRL exhibited altered chromatin profiles following shock. CALCRL provides a quality marker for endothelial cells (36), which serve an important role in the development of ALI/ARDS. A strong result in peak accessibility was notable amongst endothelial cells and several other phenotypes across all feature linkage profiles presented (Figures 5–7). This appears to echo the expression profile *Calcrl* exhibited in Figures 3B, 4B, in which positive signal was detected predominantly in endothelial cells and scattered across several other phenotypes including alveolar macrophages and the mixed population of monocytes, macrophages and dendritic cells.

The deterioration of endothelial barrier function signals a hallmark characteristic of ARDS (42, 43). It is currently unclear whether this reduced accessibility near *Calcrl* in experimental shock offers potential as a clinical biomarker for this pre-disease state. The means by which such selective gene deficiencies maintain their sham profile of high accessibility also remains uncertain. Wild-type accessibility around *Calcrl* was high in sham backgrounds as noted by total feature linkages, with strong positive correlation. The strength of this correlation indicated that the relationship between transcripts detected by RNA sequencing relative to accessible genomic loci from ATAC sequencing was nearly 1:1. This indicates these regions were tightly regulating the transcription of this gene. The reduced accessibility near *Calcrl* in the wild-type hemorrhaged background is an interesting biological result. Of further importance is the outcome of maintained accessibility following hemorrhage in the context of individual checkpoint protein deficiencies. These profiles were more similar to sham results than hemorrhaged wild-type type profiles. Following sepsis modeling it was predicted that VISTA and PD-1/L1 uniquely contributed to pre-ALI priming and/or disease states given the disparate experimental sepsis mortality seen in gene deficient backgrounds relative to wild-type counterparts (30–32). Unexpectedly, all three gene deficiencies somewhat similarly featured accessible chromatin near *Calcrl*. Albeit this was less conserved between PD-L1^–/–^ samples. Further work is necessary to clarify *Calcrl* and its role in the hemorrhaged lung microenvironment. Additional functional assays in cell types of interest may validate a defined role for this gene across hemorrhaged wild-type or deficient backgrounds.

### 3.4. Adrenomedullin signaling in sepsis and acute lung injury

CALCRL serves as a receptor for the peptide adrenomedullin (AM), along with receptor activity-modifying protein 2 (RAMP2) and RAMP3 as co-receptors (44–47). Not addressed within this manuscript is calcitonin gene-related peptide (CGRP), which binds a complex of RAMP1 and CALCRL (44, 48). Experimental data has identified multiple roles for this key ligand. In rats it has been shown that delivery of adrenomedullin binding protein-1 (AMBP-1) once sepsis was initiated, along with delivery of AM peptide prior to and throughout induction slowed the shift from hyperdynamic to hypodynamic sepsis, improved survival and reduced tissue damage associated with the insult (46). Furthermore, administering AM and AMBP-1 following sepsis induction in rats reduced inflammatory indices including circulating TNF-α, IL-6, and IL-1β (47). Importantly, circulating levels of AM are increased in septic patients (49). Such conflicting outcomes of AM activity in sepsis associated pathologies begets a murky role for this peptide in disease progression.

Experimental modeling has shown that the effects of adrenomedullin are favorable for a variety of lung injury indices, including endothelial permeability (50). It has also been confirmed that the signaling from CALCRL/AM ligation and endothelial nitric oxide synthase activity is important for the resolution of experimental bronchopulmonary dysplasia associated with pulmonary hypertension, clinically seen amongst infants (51). As others have shown, this activity is important for development of the murine lymphatic vascular system (45). These results may further suggest that the CALCRL/RAMP2 signaling axis *via* adrenomedullin ligation impacts the lung vascular endothelium in clinical contexts, and possibly the associated effects of ALI. Experimental data shared here in the context of shock induced by sustained hemorrhage highlights a potential role for this activity in the pre-ALI microenvironment.

This analysis posits similarities in *Calcrl* accessibility in the hemorrhaged lung microenvironment across cellular phenotypes of coinhibitory gene deficient backgrounds. Sham conditions appeared to present a baseline of high chromatin availability near *Calcrl* relative to an apparent reduction or loss in wild-type backgrounds, characterizing a primed state or altered predisposition of cells in the lung microenvironment. This begets the development of ALI/ARDS, whereas gene deficiencies seem to maintain homeostatic levels and mitigate priming. These data support viability of *Calcrl* as a therapeutic target for such diseases. Another consideration to be made is regarding the availability of the AM peptide across each deficient background. Of note, the PD-1/L1 deficiency offers a survival benefit in experimental ALI (52, 53). This is consistent with the aforementioned results in experimental sepsis (30–32). Additional work is planned to survey across each (post-hemorrhage) gene deficient group in order to determine whether AM levels are variable, as its ability to interact with the CALCRL endothelial receptor may play a role in either the septic immune response or shock-induced ALI priming.

### 3.5. Novel regulatory elements

The implementation of such methods for chromatin landscape profiling serves as an identifier of novel regulatory sites dispersed throughout the genome. These previously undescribed loci offer insights on the means by which widely dispersed enhancers/silencers seem to influence transcription. Correlation scores provide additional clarity on the likelihood of regulatory influence imparted by these loci. The specificity provided in this analysis is at chromosomal base pair resolution, which may be used to reference commonly occurring polymorphisms or genomic abnormalities leading to pathogenesis. While complexities in deciphering these details remain, the increased ubiquity of single cell multiomics approaches continues to favor iterative and accessible options for tailored analysis. The suite of tools for enhanced visualization offered by Loupe Browser are often modified and updated, providing the field with new and improved means of identifying highly specific druggable genomic targets.

## 4. Conclusion

The primary drivers of ALI and ARDS predispositional priming have required an additional degree of resolution not previously achieved. The complex lung microenvironment is composed of various immune/non-immune phenotypes involved in the associated hypersensitive state seen after shock. The methods presented in this pilot study offer a glimpse at clarifying altered cell specific roles resultant from shock-induced priming. The two-pronged multiomics approach of RNA/ATAC sequencing at single cell resolution has provided putative molecular target and genomic loci worthy of further analysis.

The work presented here has addressed the priming response concurrent with critical illness across backgrounds deficient in several key immunotherapeutic coinhibitory targets. This has resulted in identification of interesting alterations near the gene *Calcrl* following shock, which was significantly enriched across individual lung phenotypes. The peptide adrenomedullin AM, which binds CALCRL (44–47), has been implicated in the pathogenesis of sepsis and the immune response (46, 47). These interactions have implicated the vascular endothelium as it pertains to experimental lung injury (45, 50, 51). Despite the small sample size of this project, alterations made at the chromatin level during the pre-ALI state of shock induced priming were evident. The nature by which such reduced accessibility near CALCRL might pre-dispose to ALI/ARDS elicits additional questions pertaining to pre-transcriptional chromatin modifications. High pre-shock availability of DNA segments near CALCRL and their positive correlation with its expression suggest an apparent regulatory capacity on transcription. Post-shock coinhibitory gene deficient chromatin profiles presented similar results to that of pre-shock wild-type samples, suggesting an influence on CALCRL accessibility. Key changes illustrated in the pre-ALI context of hypovolemic/hemorrhagic shock may allow for additional resolution of “priming” and “cellular pre-activation/pre-disposition” processes within the lung microenvironment.

## 5. Materials and methods

### 5.1. Murine backgrounds

Mice deficient in PD-1 (PD-1^–/–^) or PD-L1 (PD-L1^–/–^) were used to breed the knock-out pups (provided by Tasuku Honjo, Kyoto University, Kyoto, Japan, through Megan Sykes at Massachusetts General Hospital, Boston, MA, USA) here at Lifespan-Rhode Island Hospital. VISTA deficient mice were generated through use of the CRISPR Cas9 system also in the C57BL/6 background as previously described by Gray et al. (32). Mice were genotyped to confirm global loss of expression per relevant receptor or ligand. All mice used in experimental shock modeling were aged 8–12 weeks (12: 12-h light/dark cycle, 68–72°F, 30–70% humidity).

### 5.2. Experimental model of shock

A model of hypovolemic shock was implemented, whereby a fixed-pressure hemorrhage was utilized to achieved sustained reduced mean arterial blood pressure in male C57BL/6 murine hosts aged 8–12 weeks. This choice was made so as to maximize our ability to initially see an experimental difference in the ALI/ARDS response based on previous reports that male mice did poorer in response to these experimental stressors of shock (hemorrhage) and/or septic (CLP) challenge than pro-estrus stratified females (54, 55). In brief, an isoflurane/oxygen gas mixture was used as anesthesia under the Rhode Island Hospital IACUC approved protocol for animal safety (AWC# 5064-18 and 5054-21). Bi-lateral arteriotomies were catheterized and used to monitor blood pressure and draw blood throughout the procedure. Mice were kept in this state for a 90-min duration, during which additional blood was drawn to maintain reduced blood pressure ∼40 mmHg (±5 mmHg). This provides a standardized and effective mimic of severely injured hypovolemic patients. Immediately following this experimental insult, mice were administered a crystalloid solution of lactated ringer’s equivalent to 4× the volume of blood hemorrhaged. Sham surgeries were performed under anesthesia by which both femoral arteries were ligated. However, blood was not drawn and these serve as negative controls in this analysis.

### 5.3. Preparation of samples for sequencing libraries

An *N* = 2 was established in order to compare consistencies between chromatin alterations between biological replicates. A total of 24-h post-resuscitation, mice were euthanized and lungs were harvested. Tissue was pooled from 2 mice per treatment group/genotype for all samples prepared, and flash frozen on liquid nitrogen for 10 min. These were then stored at −80°C and shipped on dry ice to the Robert Boas Center for Genomics and Human Genetics within the Feinstein Institute for Medical Research-Northwell Health, Manhasset, NY, USA, where single cell (single nucleus) multiomics libraries from 4,000 cells/sample were prepared from lysed tissue. This was then filtered and washed, followed by nuclear 7AAD exclusion staining. These were sorted and permeabilized, then washed and counted for use in subsequent steps. The 10× Genomics Chromium Next GEM Single Cell Multiome ATAC + Gene Expression library preparation kit was implemented (Product Code 1000285). Libraries were loaded and run on a Nextseq 500.

### 5.4. Single cell multiomics analysis pipeline and unsupervised analysis

Cell Ranger ARC was utilized for all pertinent pre-processing of multiomics datasets including mouse reference genome alignment (mm10), transcript counting (33). During additional pre-processing, all sample datasets were filtered and re-clustered based on cells falling within the linear distribution cut-off range of UMI’s (unique molecular identifiers) (20–20,000) per barcode and (1–30,001) features per barcode and a maximum threshold of 5% mitochondrial reads (56) through the Loupe Browser data visualization platform (34). An unsupervised analysis was performed through which clusters from the gene expression plot were identified by patterns of positive signal detected. These profiled similarly to general expression patterns in referenced murine lung NGS (35–37) datasets and others found in the online reference BioGPS (38) (Supplementary Table 1).

### 5.5. Feature linkage assessment

Linked features provide a better understanding of the correlation between significantly enriched genes per cluster relative to altered chromatin accessibility and the likely presence of regulatory genomic elements (19). These were identified for *Calcrl* across all samples. The default settings for Cell Ranger ARC were implemented in which all resolved linked features fell within 1 Mb of *Calcrl*. The total number of links for each sample is provided in Table 1.

## Data availability statement

The data presented in this study are deposited in the Brown Digital Repository, https://doi.org/10.26300/dm01-zr98.

## Ethics statement

This animal study was reviewed and approved by the Rhode Island Hospital IACUC approved protocol for animal safety (AWC# 5064-18 and 5054-21).

## Author contributions

BA initially drafted and revised this submission. BA, CL, YC, RZ, and C-SC performed the surgical techniques. BA and CL completed the sample collection. AF and SM helped BA to complete the RNA-seq and ATAC-seq analysis. YC performed the routine genotyping and husbandry of PD-1^–/–^, PD-L1^–/–^, and VISTA^–/–^ mice. AA provided guidance throughout the conception and execution of this project. All authors reviewed this manuscript.
