# Supplementary material for: Application of single cell multiomics points to changes in chromatin accessibility near calcitonin receptor like receptor and a possible role for adrenomedullin in the post-shock lung
Source: Front Med (Lausanne). 2023 Apr 11;10:1003121. doi: 10.3389/fmed.2023.1003121 (PMC10126233; doi:10.3389/fmed.2023.1003121)

**Supplementary Data**

Sequencing data and Loupe Browser files are available at DOI: https://doi.org/10.26300/dm01-zr98

Table S1. Table displays genes used to identify clusters of cells based on positive expression displayed via T-SNE. Additional tables of differentially expressed genes per cluster/sample can be made available.

| Identity | Genes | | | | | |
| --- | --- | --- | --- | --- | --- | --- |
| Alveolar Epithelial Type II Cells | Slc34a2 | abca3 | lamp3 | sftpd |  |  |
| Alveolar Epithelial Type I Cells | Rtkn2 |  |  |  |  |  |
| Club (Clara) Cells | cyp2f2 |  |  |  |  |  |
| Endothelial Cells | calcrl | tmem100 | tek |  |  |  |
| B cells | cd79b | ebf1 |  |  |  |  |
| Monocytes/Macrophages/Dendritic Cells | chil3 | plac8 | mrc1 | c1qb | itgam | itgax |
| Alveolar Macrophages | chil3 | siglecf | ear2 |  |  |  |
| Fibroblasts | inmt | col1a2 |  |  |  |  |
| Mesothelial Cells | Fras1 | Muc16 | msln |  |  |  |
| Ciliated Cells | Spef2 | Dnah6 | foxj1 |  |  |  |
| Neutrophils | ngp |  |  |  |  |  |
| NK Cells | gzma |  |  |  |  |  |

Table S2. Depicts gene expression and peak information for wild-type sham replicate 1. Correlation scores between linked features (significantly enriched gene and accessible chromatin) are also presented. Significance for these relationships is calculated based on Cell Ranger ARC baseline settings. Specific genomic loci of accessible chromatin is detailed. Feature links are within 1 Mb.

| selected_feature | selected_feature_id | linked_feature_id | distance | correlation | significance |
| --- | --- | --- | --- | --- | --- |
| Calcrl | ENSMUSG00000059588 | chr2:84184425-84185327 | -215508 | 0.824399568 | 60.26530044 |
| Calcrl | ENSMUSG00000059588 | chr2:84072597-84073497 | -327337 | 0.746461228 | 32.44880514 |
| Calcrl | ENSMUSG00000059588 | chr2:84019630-84020444 | -380347 | 0.734579655 | 25.8539485 |
| Calcrl | ENSMUSG00000059588 | chr2:84274133-84275001 | -125817 | 0.705733379 | 30.33399171 |
| Calcrl | ENSMUSG00000059588 | chr2:85129158-85130052 | 729221 | 0.70596179 | 25.06429941 |
| Calcrl | ENSMUSG00000059588 | chr2:84276766-84277561 | -123221 | 0.689338297 | 26.95542201 |
| Calcrl | ENSMUSG00000059588 | chr2:84362700-84363550 | -37259 | 0.693547726 | 16.67589056 |
| Calcrl | ENSMUSG00000059588 | chr2:84915068-84915966 | 515133 | 0.667727045 | 12.34229305 |
| Calcrl | ENSMUSG00000059588 | chr2:84229506-84230351 | -170456 | 0.648729264 | 14.52441859 |
| Calcrl | ENSMUSG00000059588 | chr2:85135943-85136756 | 735965 | 0.645329918 | 21.01641095 |
| Calcrl | ENSMUSG00000059588 | chr2:84895214-84896177 | 495311 | 0.643396633 | 12.43183363 |
| Calcrl | ENSMUSG00000059588 | chr2:85001343-85002171 | 601373 | 0.644942735 | 9.832647841 |
| Calcrl | ENSMUSG00000059588 | chr2:84345706-84346583 | -54240 | 0.607558005 | 21.43688376 |
| Calcrl | ENSMUSG00000059588 | chr2:84936308-84937225 | 536382 | 0.589953441 | 10.10496732 |
| Calcrl | ENSMUSG00000059588 | chr2:84424838-84425744 | 24907 | 0.528268338 | 5.862794789 |
| Calcrl | ENSMUSG00000059588 | chr2:84077178-84078027 | -322782 | 0.50846094 | 5.848693001 |
| Calcrl | ENSMUSG00000059588 | chr2:84239774-84240604 | -160195 | 0.452711172 | 8.300174552 |
| Calcrl | ENSMUSG00000059588 | chr2:85098596-85099510 | 698669 | 0.432186301 | 6.755782042 |

Table S3. Depicts feature linkage information for wild-type sham replicate 2, as in Table 1, which includes correlation scores and significance.

| selected_feature | selected_feature_id | linked_feature_id | distance | correlation | significance |
| --- | --- | --- | --- | --- | --- |
| Calcrl | ENSMUSG00000059588 | chr2:84184445-84185346 | -215489 | 0.802692377 | 44.70188145 |
| Calcrl | ENSMUSG00000059588 | chr2:84362596-84363500 | -37336 | 0.77539928 | 23.74650778 |
| Calcrl | ENSMUSG00000059588 | chr2:85129300-85130216 | 729374 | 0.781407399 | 60.48230353 |
| Calcrl | ENSMUSG00000059588 | chr2:84019576-84020416 | -380388 | 0.762017118 | 31.82004157 |
| Calcrl | ENSMUSG00000059588 | chr2:84274070-84274917 | -125891 | 0.753874982 | 18.19942121 |
| Calcrl | ENSMUSG00000059588 | chr2:84072618-84073485 | -327333 | 0.744027815 | 30.25369105 |
| Calcrl | ENSMUSG00000059588 | chr2:84328517-84329412 | -71420 | 0.721357453 | 36.35566144 |
| Calcrl | ENSMUSG00000059588 | chr2:84065558-84066464 | -334373 | 0.647353869 | 9.661602182 |
| Calcrl | ENSMUSG00000059588 | chr2:84424890-84425778 | 24950 | 0.636421602 | 20.59179883 |
| Calcrl | ENSMUSG00000059588 | chr2:84322029-84322980 | -77880 | 0.629999594 | 8.743564547 |
| Calcrl | ENSMUSG00000059588 | chr2:85098548-85099478 | 698629 | 0.595924288 | 12.2529494 |
| Calcrl | ENSMUSG00000059588 | chr2:85135910-85136813 | 735977 | 0.57935207 | 13.02321852 |
| Calcrl | ENSMUSG00000059588 | chr2:85238039-85239036 | 838153 | 0.557374922 | 6.308738397 |
| Calcrl | ENSMUSG00000059588 | chr2:84064245-84065039 | -335742 | 0.531066805 | 7.245322708 |
| Calcrl | ENSMUSG00000059588 | chr2:84895145-84895996 | 495186 | 0.466193674 | 9.655016831 |
| Calcrl | ENSMUSG00000059588 | chr2:84936354-84937174 | 536380 | 0.452617527 | 5.286331728 |
| Calcrl | ENSMUSG00000059588 | chr2:84229248-84230280 | -170620 | 0.427868943 | 12.00628404 |

Table S4. Depicts feature linkage information for wild-type hem replicate 1, as in Table 1, which includes correlation scores and significance.

| selected_feature | selected_feature_id | linked_feature_id | distance | correlation | significance |
| --- | --- | --- | --- | --- | --- |
| Calcrl | ENSMUSG00000059588 | chr2:84184437-84185292 | -215520 | 0.63126237 | 18.9442555 |
| Calcrl | ENSMUSG00000059588 | chr2:84424869-84425782 | 24941 | 0.45134993 | 7.18990554 |
| Calcrl | ENSMUSG00000059588 | chr2:85105424-85106302 | 705479 | 0.36730062 | 6.37902966 |

Table S5. Depicts feature linkage information for wild-type hem replicate 2, as in Table 1, which includes correlation scores and significance.

| selected_feature | selected_feature_id | linked_feature_id | distance | correlation | significance |
| --- | --- | --- | --- | --- | --- |
| Calcrl | ENSMUSG00000059588 | chr2:84065842-84066678 | -334124 | 0.44096735 | 9.51927374 |

Table S6. Depicts feature linkage information for PD-1^-/-^ hem replicate 1 as in Table 1, which includes correlation scores and significance.

| selected_feature | selected_feature_id | linked_feature_id | distance | correlation | significance |
| --- | --- | --- | --- | --- | --- |
| Calcrl | ENSMUSG00000059588 | chr2:85129175-85130087 | 729247 | 0.79864694 | 22.806103 |
| Calcrl | ENSMUSG00000059588 | chr2:84274053-84274952 | -125882 | 0.75798995 | 12.1426131 |
| Calcrl | ENSMUSG00000059588 | chr2:84938060-84938782 | 538037 | 0.71517095 | 17.6722064 |
| Calcrl | ENSMUSG00000059588 | chr2:84184420-84185286 | -215531 | 0.70062833 | 22.642378 |
| Calcrl | ENSMUSG00000059588 | chr2:85139016-85139905 | 739076 | 0.69531699 | 9.04934861 |
| Calcrl | ENSMUSG00000059588 | chr2:84065624-84066572 | -334286 | 0.67987709 | 17.6758795 |
| Calcrl | ENSMUSG00000059588 | chr2:84916601-84917765 | 516799 | 0.68470926 | 9.97665679 |
| Calcrl | ENSMUSG00000059588 | chr2:84072622-84073534 | -327306 | 0.666172 | 24.101159 |
| Calcrl | ENSMUSG00000059588 | chr2:84424885-84425782 | 24949 | 0.59375612 | 13.6212236 |
| Calcrl | ENSMUSG00000059588 | chr2:84362656-84363451 | -37331 | 0.54866059 | 15.1802881 |
| Calcrl | ENSMUSG00000059588 | chr2:84895237-84896076 | 495272 | 0.54213606 | 9.55137024 |
| Calcrl | ENSMUSG00000059588 | chr2:84936281-84937259 | 536386 | 0.52832373 | 5.15845241 |
| Calcrl | ENSMUSG00000059588 | chr2:84915044-84916001 | 515138 | 0.52064656 | 22.2253128 |
| Calcrl | ENSMUSG00000059588 | chr2:85135811-85136716 | 735879 | 0.49888161 | 8.53167302 |
| Calcrl | ENSMUSG00000059588 | chr2:85137022-85137941 | 737097 | 0.49066837 | 9.59710611 |

Table S7. Depicts feature linkage information for PD-1^-/-^ hem replicate 2 as in Table 1, which includes correlation scores and significance.

| selected_feature | selected_feature_id | linked_feature_id | distance | correlation | significance |
| --- | --- | --- | --- | --- | --- |
| Calcrl | ENSMUSG00000059588 | chr2:84072656-84073556 | -327278 | 0.64882432 | 10.9120819 |
| Calcrl | ENSMUSG00000059588 | chr2:84184280-84185347 | -215571 | 0.63443768 | 29.519673 |
| Calcrl | ENSMUSG00000059588 | chr2:84273938-84274854 | -125988 | 0.62503833 | 19.3477568 |
| Calcrl | ENSMUSG00000059588 | chr2:84362627-84363532 | -37305 | 0.62924983 | 59.4261499 |
| Calcrl | ENSMUSG00000059588 | chr2:84065651-84066588 | -334265 | 0.60510421 | 20.1585484 |
| Calcrl | ENSMUSG00000059588 | chr2:84229495-84230345 | -170464 | 0.57662166 | 9.3066583 |
| Calcrl | ENSMUSG00000059588 | chr2:84019638-84020531 | -380300 | 0.56570368 | 15.900407 |
| Calcrl | ENSMUSG00000059588 | chr2:85129192-85130093 | 729258 | 0.53609655 | 12.6564967 |
| Calcrl | ENSMUSG00000059588 | chr2:84328339-84329167 | -71631 | 0.52748523 | 9.31970431 |
| Calcrl | ENSMUSG00000059588 | chr2:84916578-84917536 | 516673 | 0.51697898 | 12.3060007 |
| Calcrl | ENSMUSG00000059588 | chr2:84424835-84425720 | 24893 | 0.50593033 | 13.0105606 |
| Calcrl | ENSMUSG00000059588 | chr2:85098542-85099476 | 698625 | 0.45428714 | 6.17141017 |
| Calcrl | ENSMUSG00000059588 | chr2:85131693-85132493 | 731709 | 0.38862912 | 5.34757529 |

Table S8. Depicts feature linkage information for PD-L1^-/-^ hem replicate 1 as in Table 1, which includes correlation scores and significance.

| selected_feature | selected_feature_id | linked_feature_id | distance | correlation | significance |
| --- | --- | --- | --- | --- | --- |
| Calcrl | ENSMUSG00000059588 | chr2:84184378-84185247 | -215572 | 0.60364329 | 12.514215 |
| Calcrl | ENSMUSG00000059588 | chr2:84072674-84073557 | -327269 | 0.5383766 | 20.1108693 |
| Calcrl | ENSMUSG00000059588 | chr2:84936230-84937227 | 536344 | 0.54164211 | 15.262389 |
| Calcrl | ENSMUSG00000059588 | chr2:85129172-85130023 | 729213 | 0.5441074 | 12.4174798 |
| Calcrl | ENSMUSG00000059588 | chr2:84273915-84274786 | -126034 | 0.51970176 | 49.084017 |
| Calcrl | ENSMUSG00000059588 | chr2:84276868-84277775 | -123063 | 0.48319003 | 5.46865837 |
| Calcrl | ENSMUSG00000059588 | chr2:84185956-84186809 | -214002 | 0.44448666 | 11.5006061 |
| Calcrl | ENSMUSG00000059588 | chr2:84019580-84020498 | -380345 | 0.20780133 | 5.30527095 |

Table S9. Depicts feature linkage information for PD-L1^-/-^ hem replicate 2 as in Table 1, which includes correlation scores and significance.

| Selected_feature | selected_feature_id | linked_feature_id | distance | correlation | significance |
| --- | --- | --- | --- | --- | --- |
| Calcrl | ENSMUSG00000059588 | chr2:84184406-84185322 | -215520 | 0.74928035 | 30.5021679 |
| Calcrl | ENSMUSG00000059588 | chr2:84019676-84020550 | -380271 | 0.72930281 | 43.961936 |
| Calcrl | ENSMUSG00000059588 | chr2:84274059-84274964 | -125873 | 0.71723915 | 31.6437401 |
| Calcrl | ENSMUSG00000059588 | chr2:84072674-84073589 | -327253 | 0.70325368 | 36.7457128 |
| Calcrl | ENSMUSG00000059588 | chr2:85129193-85130124 | 729274 | 0.70360776 | 27.8052488 |
| Calcrl | ENSMUSG00000059588 | chr2:84065857-84066635 | -334138 | 0.68611021 | 17.3667876 |
| Calcrl | ENSMUSG00000059588 | chr2:84916699-84917583 | 516757 | 0.66567904 | 16.9469518 |
| Calcrl | ENSMUSG00000059588 | chr2:85001364-85002294 | 601445 | 0.67288425 | 11.8003759 |
| Calcrl | ENSMUSG00000059588 | chr2:84424873-84425786 | 24945 | 0.59647512 | 20.9473036 |
| Calcrl | ENSMUSG00000059588 | chr2:84229466-84230303 | -170500 | 0.59448634 | 15.1930971 |
| Calcrl | ENSMUSG00000059588 | chr2:84276791-84277551 | -123213 | 0.57877379 | 18.319839 |
| Calcrl | ENSMUSG00000059588 | chr2:84322144-84323024 | -77800 | 0.55558993 | 9.83735683 |
| Calcrl | ENSMUSG00000059588 | chr2:84999170-85000097 | 599249 | 0.54354774 | 11.7121768 |
| Calcrl | ENSMUSG00000059588 | chr2:84362697-84363585 | -37243 | 0.52346153 | 15.5650866 |
| Calcrl | ENSMUSG00000059588 | chr2:84895206-84896184 | 495311 | 0.48804232 | 7.52651758 |
| Calcrl | ENSMUSG00000059588 | chr2:84383379-84384108 | -16641 | 0.4783364 | 13.0575941 |
| Calcrl | ENSMUSG00000059588 | chr2:84912397-84913243 | 512436 | 0.45286975 | 7.20901499 |
| Calcrl | ENSMUSG00000059588 | chr2:85106836-85107619 | 706843 | 0.4454727 | 8.56360148 |
| Calcrl | ENSMUSG00000059588 | chr2:85139026-85139930 | 739094 | 0.40618465 | 5.67332699 |
| Calcrl | ENSMUSG00000059588 | chr2:84459574-84460357 | 59581 | 0.36515986 | 7.42563941 |
| Calcrl | ENSMUSG00000059588 | chr2:84355204-84355774 | -44895 | 0.34802337 | 6.0290798 |
| Calcrl | ENSMUSG00000059588 | chr2:85098545-85099468 | 698622 | 0.33322419 | 5.69851747 |

Table S10. Depicts feature linkage information for VISTA^-/-^ hem replicate 1 as in Table 1, which includes correlation scores and significance.

| selected_feature | selected_feature_id | linked_feature_id | distance | correlation | significance |
| --- | --- | --- | --- | --- | --- |
| Calcrl | ENSMUSG00000059588 | chr2:84184416-84185293 | -215530 | 0.74210566 | 30.3210455 |
| Calcrl | ENSMUSG00000059588 | chr2:84274086-84275005 | -125839 | 0.72665609 | 24.9501912 |
| Calcrl | ENSMUSG00000059588 | chr2:84362668-84363474 | -37313 | 0.7167108 | 13.7860941 |
| Calcrl | ENSMUSG00000059588 | chr2:85129346-85130136 | 729357 | 0.67143451 | 14.6091905 |
| Calcrl | ENSMUSG00000059588 | chr2:84065697-84066561 | -334255 | 0.6203105 | 11.0610298 |
| Calcrl | ENSMUSG00000059588 | chr2:84072749-84073612 | -327204 | 0.5745762 | 13.9342916 |
| Calcrl | ENSMUSG00000059588 | chr2:84185988-84186810 | -213985 | 0.54225997 | 15.1725134 |
| Calcrl | ENSMUSG00000059588 | chr2:84328557-84329502 | -71355 | 0.42312517 | 5.33452694 |
| Calcrl | ENSMUSG00000059588 | chr2:84322073-84322833 | -77931 | 0.40156342 | 5.76183359 |
| Calcrl | ENSMUSG00000059588 | chr2:84002989-84003988 | -396896 | 0.29394982 | 5.32201203 |

Table S11. Depicts feature linkage information for VISTA^-/-^ hem replicate 2 as in Table 1, which includes correlation scores and significance.

| selected_feature | selected_feature_id | linked_feature_id | distance | correlation | significance |
| --- | --- | --- | --- | --- | --- |
| Calcrl | ENSMUSG00000059588 | chr2:85129230-85130155 | 729308 | 0.81662129 | 34.213773 |
| Calcrl | ENSMUSG00000059588 | chr2:84274002-84274918 | -125924 | 0.77068024 | 23.5968083 |
| Calcrl | ENSMUSG00000059588 | chr2:84072700-84073604 | -327232 | 0.74824307 | 22.95184 |
| Calcrl | ENSMUSG00000059588 | chr2:84184405-84185279 | -215542 | 0.72620508 | 31.3209673 |
| Calcrl | ENSMUSG00000059588 | chr2:84276893-84277770 | -123053 | 0.66312697 | 10.3161078 |
| Calcrl | ENSMUSG00000059588 | chr2:84322170-84323046 | -77776 | 0.65039704 | 16.3109947 |
| Calcrl | ENSMUSG00000059588 | chr2:84185889-84186794 | -214043 | 0.63007519 | 15.7934542 |
| Calcrl | ENSMUSG00000059588 | chr2:85001352-85002213 | 601398 | 0.63383275 | 14.5789018 |
| Calcrl | ENSMUSG00000059588 | chr2:84019435-84020440 | -380447 | 0.60656587 | 7.40679605 |
| Calcrl | ENSMUSG00000059588 | chr2:84229522-84230407 | -170420 | 0.60702758 | 11.402063 |
| Calcrl | ENSMUSG00000059588 | chr2:84362667-84363502 | -37300 | 0.5969375 | 13.0862084 |
| Calcrl | ENSMUSG00000059588 | chr2:84064087-84064969 | -335856 | 0.55556957 | 6.61901061 |
| Calcrl | ENSMUSG00000059588 | chr2:84424856-84425771 | 24929 | 0.54044027 | 11.5141311 |
| Calcrl | ENSMUSG00000059588 | chr2:84895231-84896130 | 495296 | 0.52392198 | 8.25989706 |
| Calcrl | ENSMUSG00000059588 | chr2:84936302-84937135 | 536334 | 0.52298905 | 5.6124458 |
| Calcrl | ENSMUSG00000059588 | chr2:85098550-85099490 | 698636 | 0.48055572 | 6.67943101 |

Figure S1. Depicts a heat map of top significant genes upregulated per cluster (log2 fold change) in wild-type sham replicate 1.


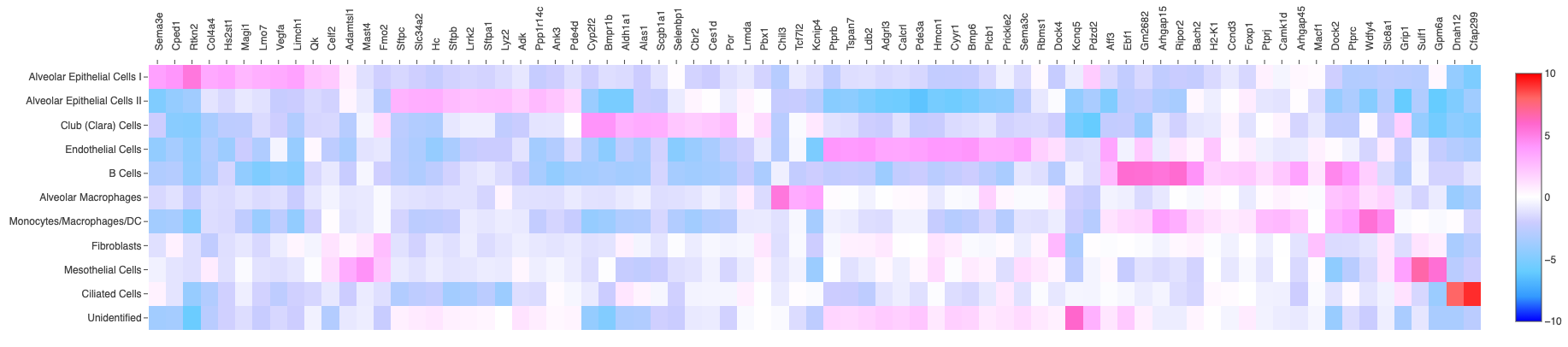


Figure S2. Depicts a heat map of top significant genes upregulated per cluster (log2 fold change) in wild-type sham replicate 2.


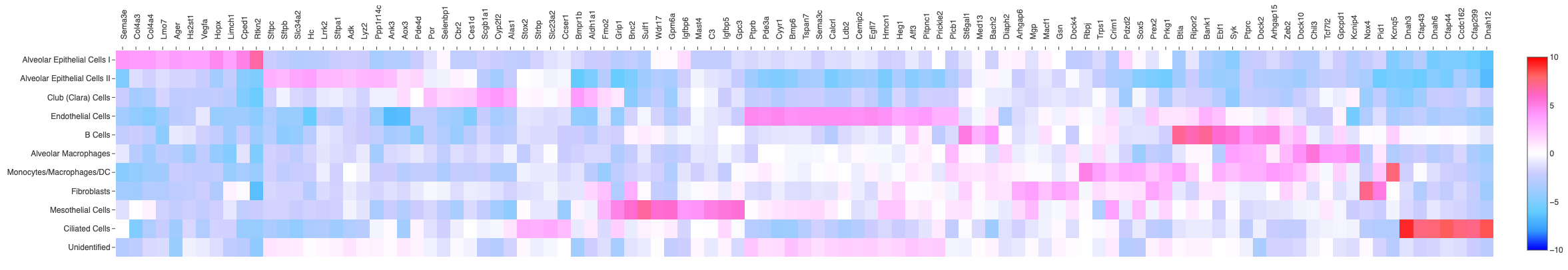


Figure S3. Depicts a heat map of top significant genes upregulated per cluster (log2 fold change) in wild-type hem replicate 1.


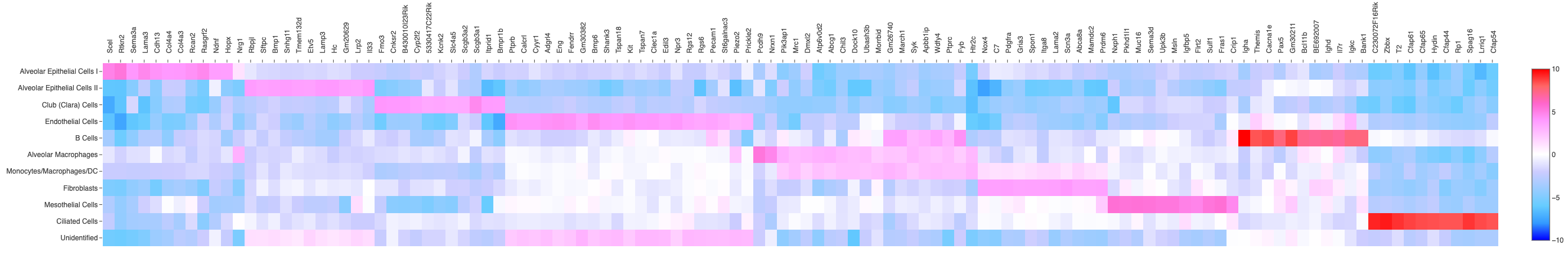


Figure S4. Depicts a heat map of top significant genes upregulated per cluster (log2 fold change) in wild-type hem replicate 2.


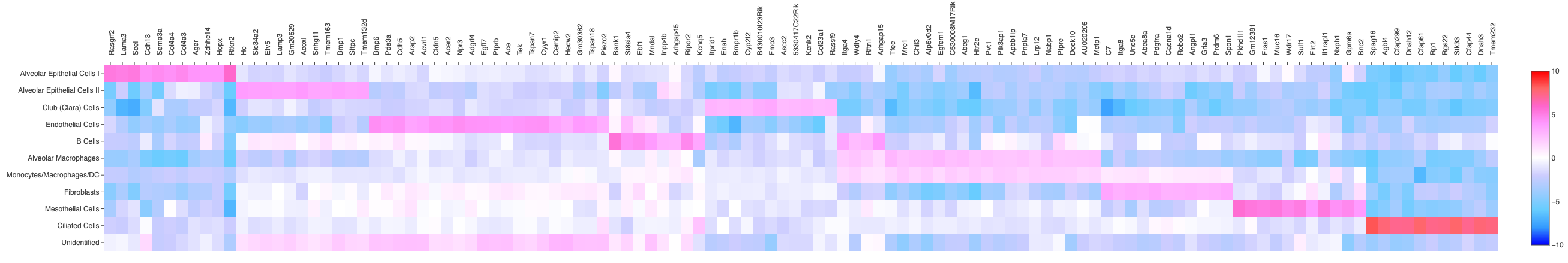


Figure S5. Depicts a heat map of top significant genes upregulated per cluster (log2 fold change) in PD-1^-/-^ hem replicate 1.


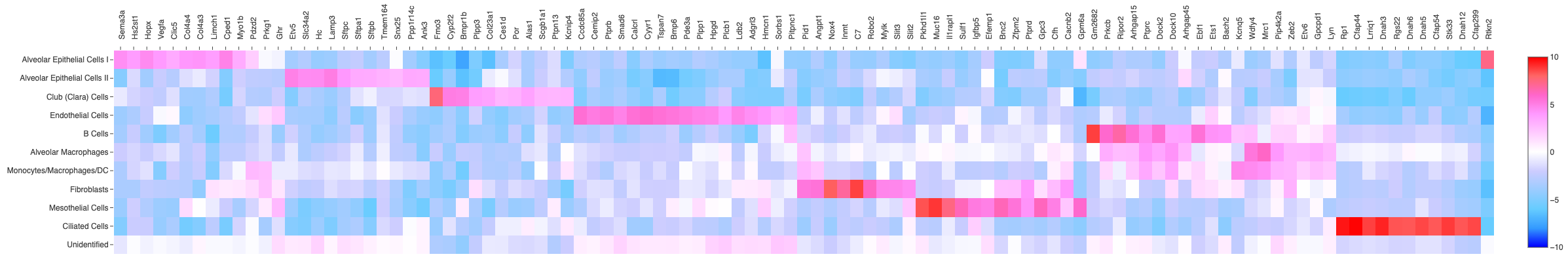


Figure S6. Depicts a heat map of top significant genes upregulated per cluster (log2 fold change) in PD-1^-/-^hem replicate 2.


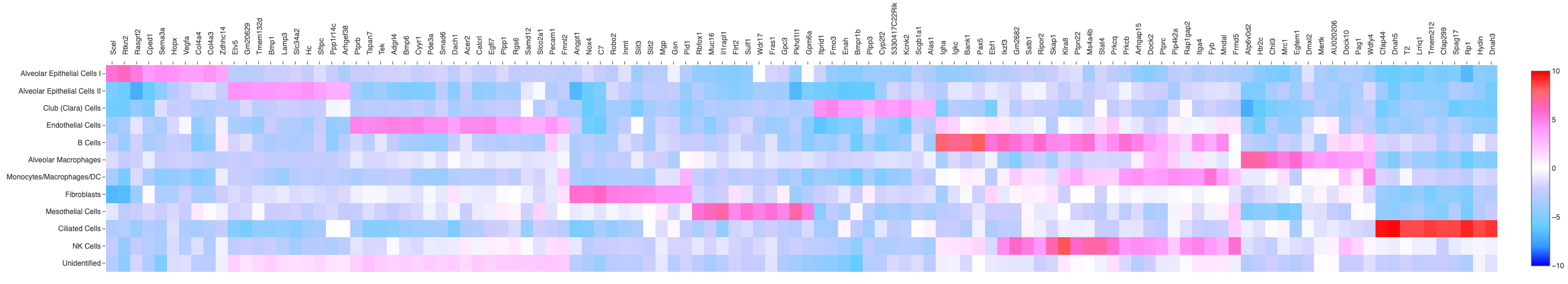


Figure S7. Depicts a heat map of top significant genes upregulated per cluster (log2 fold change) in PD-L1^-/-^hem replicate 1.


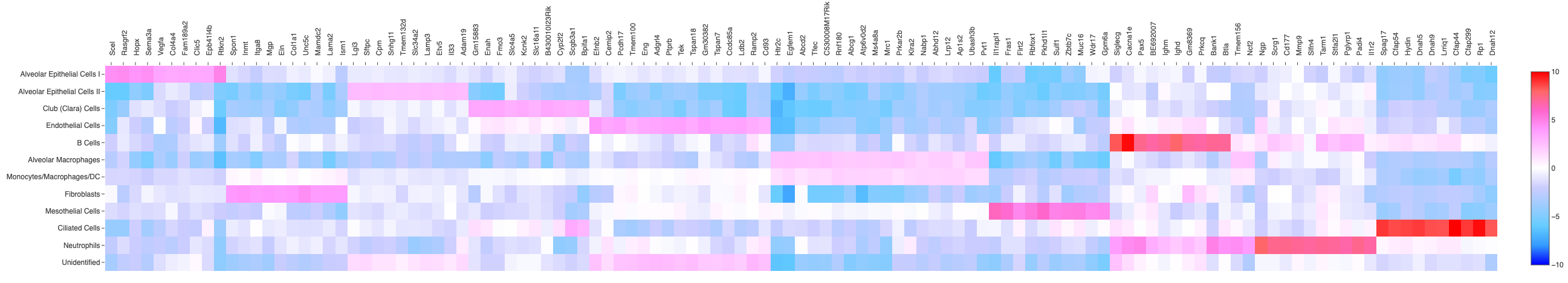


Figure S8. Depicts a heat map of top significant genes upregulated per cluster (log2 fold change) in PD-L1^-/-^hem replicate 2.


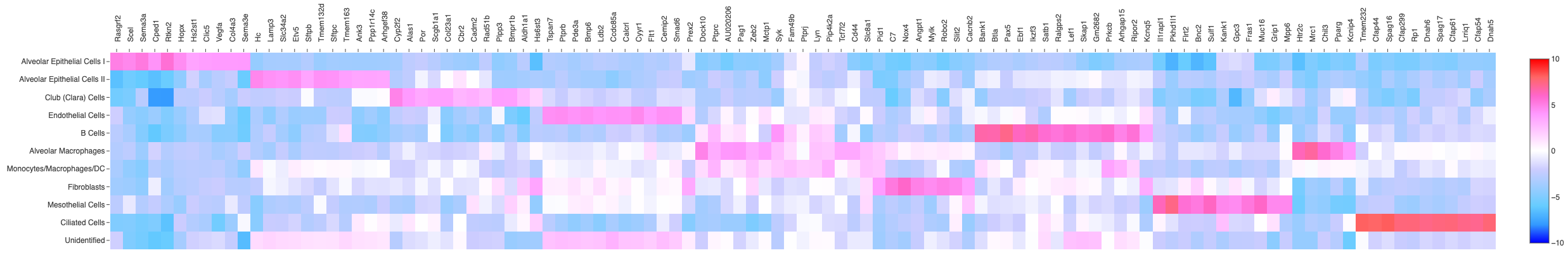


Figure S9. Depicts a heat map of top significant genes upregulated per cluster (log2 fold change) in VISTA^-/-^hem replicate 1.


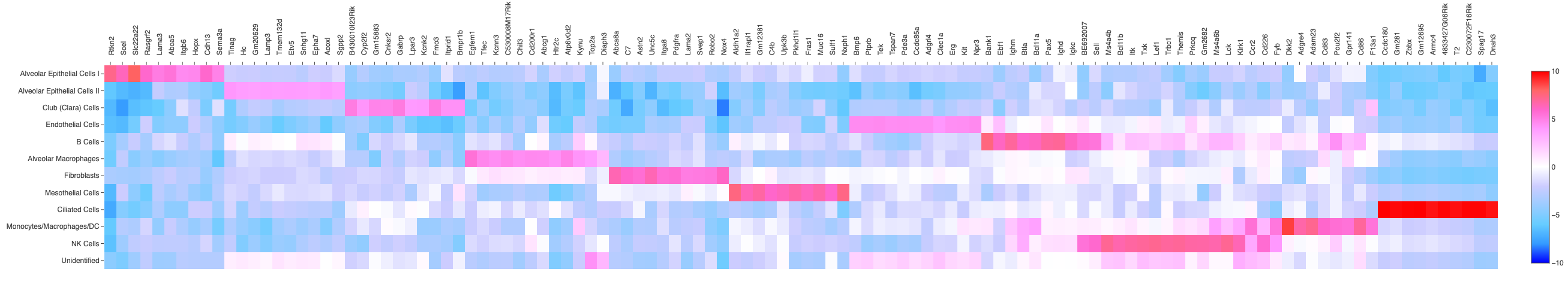


Figure S10. Depicts a heat map of top significant genes upregulated per cluster (log2 fold change) in VISTA^-/-^hem replicate 2.


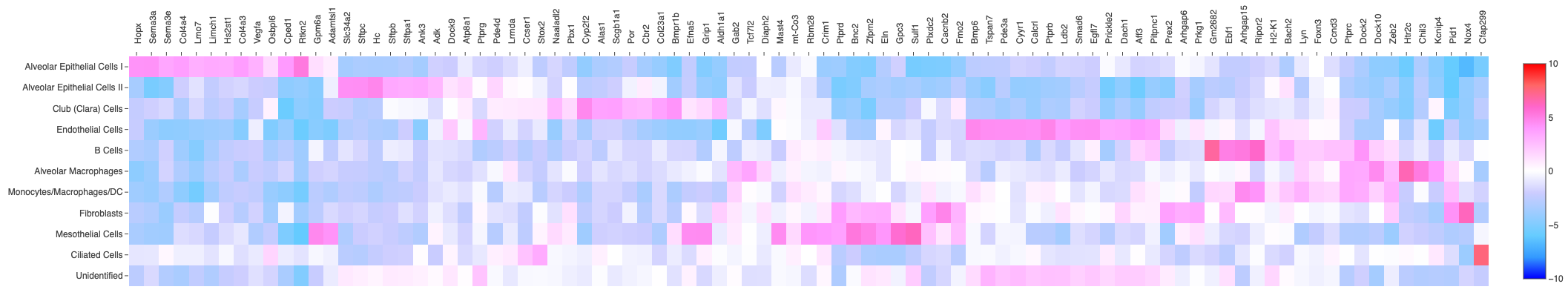

Supplement: Supplementary file 1 [file Data_Sheet_1.docx]
